# Microalternância da Onda T na Cardiomiopatia Hipertrófica: A Complexidade de uma Doença Genética com Múltiplas Expressões Fenotípicas

**DOI:** 10.36660/abc.20230615

**Published:** 2023-10-16

**Authors:** Jorge Elias

**Affiliations:** 1 Vitória Apart Hospital Serra ES Brasil Vitória Apart Hospital – Serviço de Eletrofisiologia, Serra, ES – Brasil; 2 Hospital Universitário Cassiano Antonio Moraes Ufes Vitória ES Brasil Hospital Universitário Cassiano Antonio Moraes (Hucam) – Ufes, Vitória, ES – Brasil

**Keywords:** Cardiomiopatia Hipertrófica, Morte Súbita, Arritmias Cardíacas, Desfibriladores Implantáveis

O fato de a taquiarritmia ventricular ser a causa mais frequente de morte súbita cardíaca (MSC) nas cardiomiopatias (CM) não significa que todos as CM apresentem alterações estruturais e funcionais equiparáveis e que os resultados de exames complementares, por exemplo, a análise de alterações na despolarização e repolarização ventricular, podem colaborar, de maneira semelhante, na prevenção primária da MSC.

Na edição atual do ABC Cardiol, Antunes et al.^1^ revelam que a microalternância da onda T (MAOT) não está associada à MSC e/ou arritmias ventriculares malignas em pacientes com cardiomiopatia hipertrófica (CMH).^1^ Isso sugere que os métodos diagnósticos usados para estratificação da cardiomiopatia não isquêmica (CMNI) podem não ser aplicados universalmente em um conjunto heterogêneo de patologias.

Sabe-se há muito tempo que alterações na repolarização ventricular sinalizam um alto risco de arritmias ventriculares malignas e podem servir como um marcador de risco não invasivo para MSC.^2^ Pastore et al.^3^ foram os primeiros a estabelecer uma ligação direta entre a MAOT e o início da reentrada ventricular,^3^ contribuindo para a aprovação da MAOT pelo FDA como um método não invasivo para avaliar as necessidades de implantação de CDI em diversas cardiomiopatias.^4^

A Sociedade Internacional de Holter e Eletrocardiologia Não Invasiva (ISH-NIE) recomenda a avaliação da MAOT quando há suspeita de vulnerabilidade a arritmias cardíacas letais. No entanto, nenhum dos estudos prospectivos analisados mencionou especificamente a CMH.^5^

Estudos realizados em pacientes com CMH, em geral com amostras pequenas e curto seguimento clínico, sinalizaram correlação entre a presença de MAOT e maior grau de desarranjo miofibrilar;^6^ maior associação com achados ecocardiográficos e densidade de arritmias ventriculares e heterogeneidade de locais de fibrose^7^ e com ocorrência de nVT^8^ mas não podendo se mostrar preditor de eventos arrítmicos graves.

Estudos ainda maiores tiveram um número limitado de pacientes com CMH,^9^ como o ALPHA TRIAL, que teve como foco principal a cardiomiopatia dilatada idiopática.^10^ Apesar disso, os autores seguem a mesma abordagem simplista e dicotômica e recomendam a incorporação da avaliação da MAOT nos critérios terapêuticos do CDI para cardiomiopatia não isquêmica (CMNI) classe funcional II/III da NYHA.^10^

No entanto, o presente estudo mostra que a MAOT não é um preditor confiável de eventos fatais em pacientes com CMH. Isso se deve principalmente à baixa taxa de eventos da doença e, principalmente, por ser caracterizada por heterogeneidade morfológica, funcional, clínica e prognóstica, na qual a demonstração de novos marcadores de risco (e independentes dos convencionais) é uma tarefa desafiadora.^11^

Outro ponto de interesse é a suspensão ou não da medicação betabloqueadora antes da análise da MAOT. O ISH-NIE recomenda a realização de testes de MAOT sem alterar os regimes de medicação para garantir que os resultados dos testes reflitam os efeitos da terapia medicamentosa crônica.^5^

É importante observar que os estudos sobre MAOT têm variado em seus protocolos em relação à suspensão da terapia com betabloqueadores, apesar das evidências de que esses medicamentos afetam a amplitude da MAOT e a presença de MAOT durante os testes.^12^

A frequência cardíaca não é o único determinante da MAOT porque os neurotransmissores autonômicos e as alterações no substrato miocárdico podem levar a níveis elevados de MAOT durante a estimulação de frequência fixa.^5^ Na metanálise de 9 estudos prospectivos em pacientes de prevenção primária com disfunção ventricular esquerda, o poder preditivo da MAOT variou amplamente com base na suspensão da terapia com betabloqueadores antes de sua avaliação. Os autores propuseram que esta observação pode explicar os resultados inconsistentes dos estudos de MAOT nesta população.^12^

O achado significativo do presente estudo foi que a MAOT alterada esteve associada aos critérios da AHA considerados de alto risco sem que isso se refletisse no poder de predição do desfecho primário. Os autores sinalizaram corretamente que na CMH outros mecanismos arritmogênicos, não adequadamente detectáveis por valores elevados de MAOT, estão envolvidos na origem das arritmias ventriculares e da MSC.^1^

O resultado do presente estudo corrobora uma observação que se tornou mais relevante nos últimos anos com o melhor entendimento da fisiopatologia da CMNI, achados de estudos in vivo e experimentais, resultados de exames de imagem (ressonância magnética) e, por fim, avanços na pesquisa genética.^13,14^

As observações acima são particularmente aplicáveis quando se trata de CMH. Um exemplo desta complexidade é a forma apical da CMH, que é uma expressão fenotípica clássica ligada a um bom prognóstico clínico, mas que, nas suas formas ditas atípicas (por exemplo, associadas ao aneurisma apical), apresenta um prognóstico clínico mais reservado.^13-15^ Assim, embora cada subtipo de CMH seja definido pelo seu principal fenótipo morfofuncional, uma avaliação clínica criteriosa demonstra alta variabilidade fenotípica com as devidas implicações prognósticas e terapêuticas.^14^ Outro aspecto importante é que a CMH é uma doença com expressão fenotípica variável que pode progredir e mudar no mesmo paciente.^16^

Esse achado reflete que a complexidade do substrato arritmogênico da CMH decorre de uma combinação de alterações e arranjo de miócitos, anormalidades microvasculares, fibrose intersticial/de substituição, modulação autonômica e possivelmente mutações patogênicas no gene do sarcômero. No entanto, o papel das variantes sarcoméricas como preditor de MSC ainda precisa ser demonstrado.^11,16,17^

Nesta era da medicina de precisão, o futuro é uma grande promessa para uma melhor estratificação de risco de cardiomiopatias. Possivelmente, caminharemos na direção de que, mais do que a análise de alterações na despolarização ventricular (por exemplo, fragmentação do QRS) ou na repolarização ventricular (por exemplo, MAOT), as respostas virão da detecção de alterações funcionais, estruturais e morfológicas alterações, biomarcadores genéticos e análise de potenciais fatores epigenéticos.^15,18^ Esses dados, combinados com marcadores clínicos, em um novo espaço, o da correlação fenótipo-genótipo, permitiram individualizar o risco do paciente e compartilhar a tomada de decisão quanto à necessidade ou não de um implante de CDI para prevenção primária da MSC.
