# Supplementary material for: Microalternância da Onda T na Cardiomiopatia Hipertrófica: A Complexidade de uma Doença Genética com Múltiplas Expressões Fenotípicas
Source: Arq Bras Cardiol. 2023 Oct 16;120(8):e20230615. [Article in Portuguese] doi: 10.36660/abc.20230615 (PMC10586819; doi:10.36660/abc.20230615)
Supplement: Translation

# Translation: T-Wave Microalternans in Hypertrophic Cardiomyopathy: The Complexity of an Inherited Cardiac Condition with Multiple Phenotypic Expressions

**Authors:** Jorge Elias

**Affiliations:** 1 Brazil Vitória Apart Hospital – Serviço de Eletrofisiologia, Serra, ES – Brazil; 2 Brazil Hospital Universitário Cassiano Antonio Moraes (Hucam) – Ufes, Vitória, ES – Brazil

**Keywords:** Cardiomiopatia Hipertrófica, Morte Súbita, Arritmias Cardíacas, Desfibriladores Implantáveis

The fact that ventricular tachyarrhythmia is the most frequent cause of sudden cardiac death (SCD) in cardiomyopathies (CM) does not mean that all CM exhibit similar structural and functional alterations that, identified in advance, for example, by analyzing changes in ventricular depolarization and repolarization, may collaborate in the primary prevention of SCD.

In the current edition of ABC Cardiol, Antunes et al.^1^ reveals that altered T-wave alternans (TWA) is not associated with SCD and/or malignant ventricular arrhythmias in hypertrophic cardiomyopathy (HCM) patients.^1^ This suggests that diagnostic methods used for non-ischemic cardiomyopathies (NICM). stratification may not universally apply across a heterogeneous set of pathologies.

Electrical alterations in ventricular repolarization have long been known to signal a high risk of malignant ventricular arrhythmias and could serve as a non-invasive risk marker for SCD.^2^ Pastore et al. were the first to establish a direct link between TWA and the onset of ventricular reentry,^3^ contributing to the FDA’s approval of TWA as a non-invasive method for evaluating ICD implantation needs in various cardiomyopathies.^4^

The International Society for Holter and Non-invasive Electrocardiology (ISH-NIE) recommends TWA evaluation when suspected of vulnerability to lethal cardiac arrhythmias. However, none of the prospective studies they analyzed mentioned HCM specifically.^5^

Studies carried out in patients with HCM, in general with small samples and a short clinical follow-up, signaled a correlation between the presence of TWA and a higher degree of myofibrillar disarrangement;^6^ a greater association with echocardiographic findings and ventricular arrhythmia density and heterogeneity of fibrosis sites^7^ and with the occurrence of nVT^8^ but not being able to prove to be a predictor of serious arrhythmic events.

Even larger studies have had limited HCM patient numbers,^9^ such as the ALPHA TRIAL, which primarily focused on idiopathic dilated cardiomyopathy.^10^ Despite this, the authors follow the same simplistic and dichotomous approach and recommend incorporating TWA evaluation into the ICD therapy criteria for NYHA functional class II/III NICM.^10^

However, the present study shows that TWA is not a reliable predictor of fatal events in HCM patients. This is primarily due to the disease’s low event rate and, principally, for being characterized by morphological, functional, clinical, and prognostic heterogeneity in which demonstrating new (and independent of conventional) risk markers is a challenging task.^11^

Another point of interest is whether or not to discontinue beta-blocker medication before TWA analysis. The ISH-NIE recommends performing TWA tests without altering medication regimens to ensure that test results reflect the effects of chronic drug therapy.^5^

It is important to note that studies on TWA have varied in their protocols regarding the suspension of beta-blocker therapy despite evidence that these drugs affect TWA amplitude and the presence of TWA during testing.^12^

Heart rate is not the sole determinant of TWA because autonomic neurotransmitters and changes in myocardial substrate can lead to elevated levels of TWA during fixed rate pacing.^5^ Meta-analysis of 9 prospective studies in primary prevention patients with left ventricular dysfunction, the predictive power of TWA varied widely based on whether beta-blocker therapy was withheld prior to its assessment. The authors proposed that this observation may explain the inconsistent results of TWA studies in this population.^12^

The significant finding of the present study was that altered TWA was associated with the AHA criteria considered high risk without this being reflected in the power to predict the primary outcome. The authors properly signaled that in HCM, other arrhythmogenic mechanisms, not adequately detectable by high TWA values, are involved in the origin of ventricular arrhythmias and SCD.^1^

The result of the present study corroborates an observation that has become more relevant in recent years with a better understanding of the pathophysiology of NICM, findings from in-vivo and experimental studies, results of imaging tests (magnetic resonance imaging), and, finally, advances in genetic research.^13,14^

The above observations are particularly applicable when dealing with HCM. The apical form of HCM is an example of this complexity, which is a classic phenotypic expression linked to a good clinical prognosis but which, in its so-called atypical forms (e.g., associated with apical aneurysm), presents a more reserved clinical prognosis.^13-15^ Thus, although each subtype of HCM is defined by its main morphofunctional phenotype, a careful clinical evaluation demonstrates high phenotype variability with due prognostic and therapeutic implications.^14^ Another important aspect is that HCM is a disorder with variable phenotype expression that may progress and change in the same patient.^16^

This finding reflects that the complexity of the HCM arrhythmogenic substrate stems from a combination of alterations and arrangement of myocytes, microvascular abnormalities, interstitial/replacement fibrosis, autonomic modulation, and possibly pathogenic sarcomere gene mutations. However, the role of sarcomeric variants as a predictor of SCD remains to be demonstrated.^11,16,17^

In this era of precision medicine, the future holds great promise for improved risk stratification of cardiomyopathies. Possibly, we will move in the direction that, more than the analysis of changes in ventricular depolarization (e.g., fragmentation of the QRS) or ventricular repolarization (e.g., TWA), the answers will come from the detection of functional, structural, and morphological alterations, genetic biomarkers and analysis of potential epigenetic factors^15,18^ These data, combined with clinical markers, in a new space, that of the phenotype-genotype correlation, allowed individualizing the patient’s risk and shared decision-making regarding the need or not for an ICD implant for the primary prevention of SCD.
